# What Is Meant by Nervous Prostration

**Published:** 1884-03

**Authors:** Roberts Bartholow

**Affiliations:** Professor of Materia Medica and General Therapeutics, in the Jefferson Medical College, of Philadelphia


### What is Meant by Nervous Prostration.

Read before the Philadelphia County Medical Society, December 19th, 1883.

BY ROBERTS BARTHOLOW, M. D., LL. D.

Professor of Materia Medica and General Therapeutics, in the Jefferson Medical College, of Philadelphia.

The popular conception of the condition now known as "nervous prostration" is a state of debility, in which nervous derangements predominate. A man actively engaged in business or in public life presently finds himself unequal to his daily tasks; he suffers odd sensations in his head; his digestion is disordered: he is weak; wakefulness, mental depression, and a thousand and one new sensations of strange character and fearful portent are superadded. The unfortunate subject of these ills now recoils from his work, gives himself up to the consideration of his symptoms, and relaxes his hold on the interests and occupations of his life. All the world declares that he has "nervous prostration," and this explanation satisfies. Physicians say "neurasthenia" or "hypochondria," according to their habits of mind or to their training. Sometimes this condition is called the "American Disease." Indeed, there is a general notion, widely prevalent, that neurasthenia is a peculiarly American malady. The late Dr. Beard was the apostle of this dispensation, and he not only was noisy and persistent in his advocacy of that view, but claimed, indeed, to have first clearly defined neurasthenia, and to have classified under this designation the numerous symptoms pertaining thereto. If we cannot admit Dr. Beard's claim in its entirety, if we experience repulsion at his tremendous but unconscious egotism, we are still compelled to acknowledge that his work in this connection is the most important that has appeared. He was peculiarly fitted to differentiate this malady, by reason of the quickness and acuteness of his intellect, his power of analysis in its subtlest aspects, and his far-reaching, his omnivorous faculty for related facts.

The term *neurasthenia*, advocated by Beard, is by no means of recent origin. The corresponding French word, used in the same sense as we now employ it, has been a stock word of French neurological medicine for fifty years. Under the terms spinal irritation, hysteria hypochondriasis, the nervous state, etc., symptoms of the same character as those now included in the word neurasthenia have been described. Besides the general state, similar derangements of functions of particular organs have been separately considered, as palpitation of the heart, headache, flatulence, impotence, etc. In the word neurasthenia—popularly, nervous prostration—the whole morbid complexus is included. The question I have to consider is whether this is real, a substantive disorder. Are the notions now generally entertained about it founded on a true conception of the condition?

I need not enlarge on the importance of a correct understanding of a morbid state which is supposed to be due to the conditions of modern, especially of American, life. Without stopping now to question the soundness of the prevailing doctrine, I will place before you the clinical history of two cases, representatives of the two types of neurasthenia. These may be designated respectively as the *congestive* and the *anæmic* varieties. The latter are greatly more numerous, but the former are not uncommon, as Beard admits.

### CASE I.—-THE CONGESTIVE TYPE.

Mr. ——, æt. 44, president of one of the largest railroad corporations of the West. He is now a robust man, 5 feet 10 inches in height, 196 pounds in weight, and has a very dark complexion, his type of constitution being the so-called bilio-nervo-sanguineous. Beginning his career at an early age, in a subordinate position, he has, by the force of a superior intellect, and of a physique that no labor could subdue, risen to the highest office, and now controls vast interests. Ambitious, enterprising, resolute, he has carried these faculties into all his work, and has shrunk from no tasks, however severe—from no responsibility, however onerous. As he has risen in position, social engagements have also added to his burdens. His mode of life has changed, to some extent. His habits have become more sedentary, although diversified by frequent railroad journeys; the pleasures of the table, including wine-drinking and late suppers, have been more and more indulged in; excessive smoking has been added to these indulgences; and thus, whilst his physical powers have been slowly impaired by bad hygiene, the demands on his mental powers have increased. Extensive interests, uncertain, often precarious, business arrangements, and the incessant watchfulness required when vast combinations may be wrecked through failure at any point, demand the highest use of every faculty; and thus to work is added worry.

Three years ago Mr. —— observed that he was not feeling well, and that he could not work as before. He became dull, especially after meals, had a constant headache, dizziness, and throbbing of the temples; he applied his mind with difficulty, and all of the head symptoms were increased by the efforts made; he had a good, rather a keen appetite; a heavily coated tongue, flatulence, constipation, and some colic pains. The bladder was rather irritable, especially at night; sexual inclination had declined, with lessened power, and various ill-defined but annoying sensations were felt about the penis, scrotum, and perineum. During the first year the symptoms increased; the attacks of vertigo were sometimes very severe, so that he had to support himself for a moment to save him from falling. On several occasions he became very much dazed, even lost consciousness momentarily, and once wandered some distance from the proper route he was taking. Anomalous sensations of creeping and crawling, coldness, and tingling, and often a burning heat, were felt in the scalp; sudden detonation in the center of the head apparently; buzzing and singing in the ears, and very constant headache, were also experienced. In the extremities, the tongue, and the genitals, there were felt peculiar tingling, numbness, coldness, creeping, and similar sensations. During the whole time of the existence of his symptoms, Mr. —— suffered from depression of spirits, a deep melancholy, in fact, and he lived in constant apprehension of failure of mind.

Physicians whom he consulted in the West located the malady in the brain, diagnosticated cerebral hyperæmia, the prelude to softening.

When Mr. —— came to see me, sixteen months ago, the symptoms just detailed continued, and were rather increased than diminished. The objective examination furnished the following details:

His face is full, the eyelids puffy, and the lower lid swollen into a bag; the conjunctivæ are injected, the sclerotic muddy, and the pupil sluggish in movement. On opthalmoscopic examination, the fundus is seen to be injected, small vessels prominent, veins swollen. There is no optical defect, except that due to his age. The membrana tympani is also rather deeply red, and vessels too small to be seen under ordinary circumstances are now in view. Hearing is unaffected.

Motility, sensibility—the tactile, pain, and temperature senses—are unaffected; and the reflexes remain normal, although probably a little sluggish. The electrical reactions are normal.

His tongue is heavily coated, the breath foul. His appetite is good, but a sense of fullness at the epigastrium persists for several hours after meals; acidity and eructations of rather foul gas now and then occur. The stools have the normal appearance, consistence, color, and odor. The urine is copious, acid, specific gravity rather high (1.025 to 1.030), and there are traces of sugar, as is usual under such circumstances.

The action of the heart is good, the pulse regular, the tension of the vessel rather high. The respiratory movements and murmurs are normal. The area of hepatic dullness is rather enlarged, and the splenic dullness seems, also, to be increased.

Subjectively the following symptoms are experienced: Various strange sensations in the scalp; a persistent headache; blurred vision at times; vertiginous feelings occurring irregularly and of varying severity; despondency, vague apprehensions; fear of places, especially of crowded assemblages; difficulty of deciding questions, very trivial or otherwise, in place of former promptness; impaired memory for persons, names, and things.

Notwithstanding this extended list of symptoms, Mr. —— did not have an ill look, but, on the contrary, on superficial examination, appeared to be robust. To him and to his immediate family the situation seemed in a high degree alarming. The surrender of his position and his business interests was regarded as imminent. To the apprehension awakened by his head symptoms was added the diagnosis of cerebral congestion, and hence the profound melancholy into which he was plunged.

Commentary.—My conclusion was that the disturbance in the functions of the brain and nervous system were secondary to derangement of the assimilative processes—of the primary and secondary assimilation—and that to the functional disorder thus caused are added the effects of introspection, and the realization by the centers of conscious impressions to an unusual extent, of ordinary peripheral excitations. My reasons for coming to this conclusion will appear hereafter. The remedies consisted in a careful regulation of the diet, in baths, exercise, in a reduction of the hours devoted to work, but not the cessation of work; in the use of a laxative quantity of sodium phosphate daily, and in the administration of the aqueous extract of ergot, with the chloride of gold and sodium, and a minute quantity of bichloride of mercury. If time and space would allow, the details of the hygienic management—so important in these cases—could very profitably, I think, occupy our attention. But I must pass on to the next case.

### CASE II.—THE ANÆMIC TYPE.

Mr. ——, æt. 56; lawyer by profession. His type of somatic constitution is the nervo-sanguine; weight, 145; height, 5 feet 9 inches. He has immense mental energy, extraordinary quickness of perception, a capital, logical, and critical faculty, and fine oratorical power. These native abilities, conjoined with extensive cultivation, soon placed him amongst the foremost men at the bar of the city where he practiced, and have long maintained him in that position. For many years he has been a dyspeptic, and suffered much from eructations of gas, from acidity and flatulence. At times—months, even years intervening—he has experienced very severe seizures, accompanied by extreme mental depression, alternating with as extreme mental exaltation. During the past five years he has had two attacks of gout, neither severe nor protracted. During the whole course of his professional life he has sustained no reverses, encountered no other anxieties than those of a successful lawyer, and has been rather singularly free, indeed, from the worries of life. Receiving last summer the nomination as a candidate to an important office, this cultivated gentleman, scholar, and lawyer; this man of nice tastes and high tone, entered on a canvass marked by vituperation and slander to an unusual extent. About the same time some business interests became entangled, and caused no little worry. During the campaign he visited some malarious districts, and spoke several times at night, in the open air. A speaker of great readiness and power, he never suffered from any considerable fatigue after public speaking, and hence he was now surprised to find himself exceedingly tired, after even a brief effort. He began to have drenching night sweats, lost his appetite, grew weak, and was compelled to return home. It was then ascertained that he had malarial fever, and was treated accordingly. But at this time, and subsequently, symptoms not necessarily of malarial origin appeared. He became frightfully dyspeptic, had enormous eructations of gas, and very considerable flatulence; his arms and legs had a numb feeling, attended with "pins and needles;" he walked with some difficulty, partly because of weakness; he was somnolent and slept a good deal, and his spirits were extremely depressed, especially on awaking in the morning. During these periods of depression he was so overwhelmed with despondency that he was apprehensive he would lose his self-control entirely.

When he placed himself under my charge, he had still a slight daily paroxysm of fever, the exacerbation occurring in the morning; but this disappeared in a few days, under the action of some efficient doses of quinine. He was very weak, pallid, and emaciated, and slept a good deal of the time. He had no headache; his vision was rather dull, and ideas and speech slow. Every morning, on awaking, he was profoundly melancholic, and all the annoyances which the campaign had developed were gone over in his mind. He could talk of nothing else, think of nothing else, than his ill feelings and the disagreeable political and personal slanders of which he had been made the victim. He complained much of the numbness of his hands, of weakness in the limbs; and he talked incessantly of his depressed feelings. The bladder became irritable, and he was compelled to rise every two or three hours during the night, the urine being acid, and depositing heavily of uric acid. Presently the somnolence was displaced by insomnia, and he slept less and less, and rose in the morning haggard, exhausted, and horribly nervous and depressed. Ordinary hypnotics proved unequal to the effort to force sleep, and increasing doses of chloral became necessary. His mental activity, heretofore so remarkable, declined, and the effort to force his mind to the performance of any work, such as letter-writing, caused a sensation of fatigue. He also became undecided, even in small matters; ceased to have any inclination to go out and mingle with the public, and grew more and more averse to political movements. He reached a point, finally, when to meet strangers caused him great distress, excited the circulation, and induced a cold sweat.

As it became indispensable that he should resume the canvass, he made a strong effort, and notwithstanding the fatigue, mental and moral depression, and exposure of public speaking, handshaking, and other matters of political expediency, he actually improved somewhat. The insomnia, irritable bladder, and hypochondriasis, however, continued, but to a less degree. In a few weeks, by means chiefly hygienical, I succeeded in stopping the chloral; natural sleep was resumed, although it remained somewhat fitful. Suitable dietetic regulations, baths, exercise, and medicines *pro re nata*, removed, or at least greatly modified the principal symptoms. Two weeks at Atlantic City accomplished no little good, and when he returned to Philadelphia last week, he appeared to be nearly his old self.

Commentary.—In this case we have exhibited that complexus of symptoms entitled neurasthenia, or nervous prostration in its anæmic form, produced by several factors—moral and somatic. The moral were very influential, but, unless the conditions producing bodily depression had occurred, the former causes could hardly have effected such results. Long-standing dyspepsia had prepared the way; malarial intoxication and fatigue contributed an important series of changes, and upon this weakened bodily state were precipitated crushing moral influences.

These cases, whose histories I have just read, are typical; each is the representative of a group. The causes are complex; the effects are not limited to one organ, or set of organs, but involve the system in general. To name this malady from the disturbance in one system seems to me an error, unless the definition is sufficiently elastic to include all the functions affected. Neurasthenia names one, only, of the parts involved. To entitle this the "American Disease," is a strange misnomer. It might with more propriety be called the "French Disease," for a condition known as the "nervous state," as "nervism," as "neurasthenia," and similar terms, has been recognized and frequently described by French writers, from an early period in this century. In France have existed the causes in the most influential form. The frequent political convulsions, the exacting social life of the great cities, and the harassing struggle for existence, inseparable from the state of the great mass of the population, induce—if any mere external conditions can—that which is called nervous exhaustion. There are two factors supposed to be especially influential in this country—work, and our exciting political and social life. I believe that the effect of these is greatly overrated.

The brain, of all the organs of the body, illustrates, in the most perfect manner, that which has been happily styled "the principle of least action." That is, to execute given tasks, it expends the least possible force, or, to express the same idea in another form, its work is done with ease, with the minimum of effort. Given a certain amount of repose—sleep—and supplied with proper nutriment—healthy blood—the brain will do its allotted work coutinuously during its working—the waking—hours. So far from being injured by severe labor carried on under normal conditions, the brain is improved by it. Mental activity, like muscular exercise, keeps the brain in a healthy state. When, therefore, a man says he is suffering from the effects of mental overwork, I want to know what his vices are. Worry may be one of these. Worry is exhausting. The worries of life do infinitely more harm than the work of life, how onerous soever it may be. The cases I have just presented illustrate this.

I deny that life is more exciting on this side of the Atlantic. The one prize of life is money, and to get possession of it is the supreme purpose, to the attainment of which every energy is put forth. Is it less so elsewhere? Who are the peoples that despise money, and make no effort to obtain it? Here life is less exciting, because our political condition is stable, and but comparatively little exertion is required to secure a comfortable subsistence. I am speaking now of the mass of the population, and not of the few consumed by ambition for political and social distinction, or led by a pitiless greed. It is the very ease and luxury of our American life that cause mischief. It is the indulgence in eating and drinking, the abuse of alcohol and tobacco, sexual excesses, sedentary habits, and too luxurious lives generally, that induce the state of the system called nervous exhaustion. If I had time, each of these should be considered, in relation to the subject. In the first case I narrated, the pleasures of the table and disordered assimilative functions caused the trouble. In the second ease, dyspepsia, malarial toxæmia, and unusual fatigue were the pathogenic factors. In both, the effects of these causes were increased by moral influences—in one, the anxieties involved in vast business enterprises; in the other, the excitement of a hot political contest. These moral causes would have had no injurious effect, had not the somatic conditions been unfavorable.

I come now to the most difficult part of my subject. I have to answer this important question: Why are the somatic derangements caused by the conditions refered to, in some cases accompanied by the mental and nervous symptoms which belong to neurasthenia? Why do some subjects with indigestion and assimilative disorders, or with the results of dyspepsia and malaria, suffer from the derangements of the mental and nervous functions, and not others? I might here take refuge behind an accepted generalization, and say that the presence or absence of the neurotic type of constitution explained the difference in the result. There is aptness in this explanation, but it is not entirely adequate.

There is a mental condition of great importance, and unless we comprehend this, we fail to realize all the possibilities of the nervous side of these cases. I, however, barely hint at the main points, under these circumstances. Besides, I wish to avoid a too metaphysical discussion of the subject.

In the conduct of life, every man who has a position to make or to maintain, exerts a certain moral force to hold himself up to his work. Some men are so happily constituted that they are quite unconscious of the effort, and stand in the front, serenely confident. Others are all the time laboring; they feel it and know it; like the soldiers of Thomas's corps at the battle of Chickamauga, who, sorely pressed, now and then looked back to see if their grim and resolute commander was still behind them with his invincible courage. Men conscious of the effort making to keep up need but little excuse to surrender themselves to their sensations. At the present time nervous prostration is much feared; its symptomatology is a common subject of discussion; and hence, familiar with its character, a man who is arrested in his career by some of the ailments supposed to belong to it, his imagination readily supplies the rest. When a man begins the study of his bodily sensations, having a certain model in his mind, he has little difficulty in filling out the details. All the world knows that when the attention is strongly fixed on an organ of the body, functional disturbances of it ensue, and finally structural changes may be induced. No part of the body is without sensation, even in health. To perceive these sensations the attention needs to be withdrawn from external things, and concentrated on the part. Thus it is when the subject of neurasthenia pursues his introspection, he becomes conscious of numerous sensations which, because now felt for the first time, are new. Under these circumstances, also, the seat of conscious impressions becomes more acutely perceptive. Suggestion adds its quota of symptoms.

To the indefinite and multiplying nervous symptoms, developing thus subjectively, must be added the reflex. Headache, vertigo, *tinnitus aurium*, amaurosis, diplopia, hallucinations and illusions, defects of speech, paralysis, are reflex symptoms on the part of the brain; palpitation, intermittent pulse, angina, pectoris, laryngismus, stridulus, asthma, are amongst the reflexes of the respiratory organs and heart; neuralgia, anæsthesia, and other disorders of the sensory nerves, and local paralysis, affections of the motor nerves, included amongst the nerve reflexes, may all be dependent on reflex excitations proceeding from the stomach.

Indeed, there is no symptom in Beard's catalogue of those belonging to neurasthenia that may not be due to merely reflex influences having their initial seat in the digestive apparatus. It follows that the term neurasthenia, or its common equivalent, nervous prostration, is either inadequate, or it expresses too much. Inadequate, if the complex of symptoms includes the functional disturbances of all the organs affected; expresses too much if the malady is a merely nervous one.

In reply to the question: "What is meant by nervous prostration?" I respond, "a disease, usually functional, situated in one or more organs, during the course of which reflex disturbances of the brain occur, and numerous subjective sensations in all parts of the body are realized by the consciousness."

I deny that neurasthenia is a primary nervous affection, or that it is a substantive disease. I hold that it is symtomatic and secondary.

This conception fixed in the mind, the treatment of neuras thenia is successful or unsuccessful, according to the measure of our skill in localizing the initial disturbance, and in addressing our remedies to that as well as to the general state.—*The Polyclinic.*

